# Drought Exposed *Burkholderia seminalis* JRBHU6 Exhibits Antimicrobial Potential Through Pyrazine-1,4-Dione Derivatives Targeting Multiple Bacterial and Fungal Proteins

**DOI:** 10.3389/fmicb.2021.633036

**Published:** 2021-04-14

**Authors:** Jay Kishor Prasad, Priyanka Pandey, Richa Anand, Richa Raghuwanshi

**Affiliations:** ^1^Department of Botany, Institute of Science, Banaras Hindu University, Varanasi, India; ^2^Department of Chemistry, Institute of Science, Banaras Hindu University, Varanasi, India; ^3^Department of Applied Science, Indian Institute of Information Technology-Allahabad, Prayagraj, India; ^4^Department of Botany, MMV, Banaras Hindu University, Varanasi, India

**Keywords:** *Burkholderia seminalis*, antimicrobial activity, spectroscopic analysis, molecular docking, pyrazine-1, 4-dione, microbial proteins

## Abstract

The present study aimed to explore the antimicrobial potentials of soil bacteria and identify the bioactive compounds and their likely targets through *in silico* studies. A total 53 bacterial isolates were screened for their antimicrobial potential of which the strain JRBHU6 showing highest antimicrobial activity was identified as *Burkholderia seminalis* (GenBank accession no. MK500868) based on 16S ribosomal RNA (rRNA) gene sequencing and phylogenetic analysis. *B. seminalis* JRBHU6 also produced hydrolytic enzymes chitinases and cellulase of significance in accrediting its antimicrobial nature. The bioactive metabolites produced by the isolate were extracted in different organic solvents among which methanolic extract showed best growth-suppressing activities toward multidrug resistant *Staphylococcus aureus* and fungal strains, *viz Fusarium oxysporum*, *Aspergillus niger*, *Microsporum gypseum*, *Trichophyton mentagrophytes*, and *Trichoderma harzianum*. The antimicrobial compounds were purified using silica gel thin layer chromatography and high-performance liquid chromatography (HPLC). On the basis of spectroscopic analysis, the bioactive metabolites were identified as pyrrolo(1,2-a)pyrazine-1,4-dione,hexahydro (PPDH) and pyrrolo(1,2-a)pyrazine-1,4-dione, hexahydro-3(2-methylpropyl) (PPDHMP). *In silico* molecular docking studies showed the bioactive compounds targeting fungal and bacterial proteins, among which PPDHMP was multitargeting in nature as reported for the first time through this study.

## Introduction

Agriculture, globally, is getting affected by the changing weather conditions and attack by phytopathogens. To deal with the ever-growing population and to sustain a stable input in the nation’s economy, there has always been a search for an eco friendly approach to overcome phytopathogens and increase production. Biocontrol efficiency of any microbe is attributed to the wide array of bioactive compounds produced by them, which repress their competitors in the rhizosphere. The bioactive compounds are basically secondary metabolites that are not vital for microbial growth/reproduction but provides miscellaneous survival purposes in nature (Martín et al., 2005; Elshafie et al., 2017b, c) and have been exploited against the emerging multidrug resistance observed in phytopathogens (Magiorakos et al., 2012). Microbes living in profoundly competitive conditions normally produce secondary metabolites to limit their ecological competitors (Mearns-Spragg et al., 1998). Investigations on these metabolites have recently gained prominence with increasing recognitions of their origin, function, and structural diversity. The global antibiotic market in 2017 made $42.33 billion and is likely to reach by 2025 upto $49.93 billion, registering 2.1% compound annual growth rate (CAGR) from 2018 to 2025 (Anonymous, 2018). Bacteria, so far, has been and is anticipated to remain in the future the most reliable resource for antibiotic, as major drugs have been developed from the lead structures inspired from bacterial products (Elbendary et al., 2018). Microbes are the most versatile producers of bioactive compounds among which the prokaryotes dominate. Alone, Actinomycetales members produce over 10,000 bioactive compounds representing 45% of the microbial metabolites (Abdel-Razek et al., 2020).

*Burkholderia*, a ubiquitous inhabitant in soil, has been reported to suppress many soil-borne plant pathogens (Weller, 1988; Raaijmakers et al., 2002; Araújo et al., 2016; Elshafie et al., 2017a; Rojas-Rojas et al., 2018). Particularly, *Burkholderia cepacia* has been used as an efficient biocontrol agent in damping off caused by *Pythium*, root rot of pea caused by Aphanomyces (King and Parke, 1993), and root rot of *Poinsettia* caused by *Rhizoctonia solani* (Cartwright and Benson, 1995). The antagonistic behavior of *Burkholderia* has been worked out to be due to the antifungal compounds secreted in the environment like pyrrolnitrin, 2,4-diacetylphloroglucinol, pyoluteorin, and phenazine (Rosales et al., 1995). Many other bacterial strains like *Pseudomonas* have also been reported to produce various antibiotics (Parker et al., 1984; Lee et al., 1994; Elshafie et al., 2012). Natural products have higher chemical novelty than that of synthetic drugs and are proving a boon in pharmaceutical industries in hand with the molecular informatics, which has come up as an important tool in finding the relationships between molecules and their biological effects (Bender and Glen, 2004). The present study was therefore aimed to screen soil bacteria exhibiting strong antimicrobial activity, identify the bioactive compounds, elucidate the mode of action of compounds identified in antimicrobial assay using *in silico* techniques, and identify the likely targets through molecular docking studies.

## Materials and Methods

### Collection, Screening, and Identification of Bacterial Isolates

#### Collection of Soil Sample

Soil samples were collected from the drought-affected region of rhizospheric soil of Arhar [*Cajanus cajan* (L.)], maize (*Zea mays* L.), and wheat (*Triticum aestivum* L.) plants growing in different states, *viz* Uttar Pradesh (UP), Bihar, and Madhya Pradesh (MP) of India, and stored at 4°C for further analysis. The soil of the collection sites had good permeability and was silty loam in nature.

#### Screening of Bacterial Isolates

Bacteria were isolated from rhizospheric soil samples by adopting the technique of serial dilution. Required dilution was spread on yeast extract mannitol agar (YEMA) media containing per liter of distilled water: yeast extract, 1.00 g; KH_2_PO_4_, 0.50 g; mannitol, 10.00 g; MgSO_4_.7H_2_O, 0.20 g; NaCl, 0.10 g; and agar, 15.00 g, adjusting pH 6.8–7.0. Plates were kept in an incubator at 28°C for 24–48 h. Colonies were handpicked from the master plates and sustained as pure cultures in YEMA media with regular transfer to fresh media and stocked for further analysis. The isolates obtained were characterized for Gram staining, colony morphology, and biochemical characters and screened for their antimicrobial activity.

#### Molecular Identification

Among all the 53 bacterial isolates, the strain JRBHU6, screened for its potential biocontrol activity, was subjected to molecular characterization. The DNA was isolated using a spin column kit (Himedia, India). The universal bacterial primers, forward: −530F (5′-GCTCTAGA GCTGACTGACTGAGTGCCAGCMGCCGCGG-3′); reverse: −800R (5′TACCAGGGTATCTAATCC3′) were used for the amplification of 16S ribosomal RNA (rRNA) gene. The PCR was carried out in 50 μl PCR mixtures including genomic DNA (gDNA) (∼50 ng), forward primer (100 ng), reverse primer (100 ng), 10 mM deoxyribonucleotide triphosphates (dNTPs) mix (2 μl), 10 × AMTaq Pol. buffer (5 μl), and AMTaq polymerase enzyme (3 U). Cycling conditions were as follows: initial denaturation at 94°C for 5 min, 35 cycles of 94°C for 30 s, 55°C for 30 s, and 72°C for 1.30 min, and a final extension of 5 min at 72°C. Purified amplicons were sequenced by Sanger method in an ABI 3500xL genetic analyzer (Life Technologies, United States). Sequencing files (.ab1) were edited using CHROMASLITE (version 1.5) and further analyzed by Basic Local Alignment Search Tool (BLAST) with closest culture sequence retrieved from the National Centre for Biotechnology Information (NCBI) database that finds regions of local similarity between sequences. The 16S rRNA gene sequence obtained for the isolated bacterial strain JRBHU6 was compared with other bacterial sequences using NCBI mega BLASTn^1^ for their pairwise identities. Multiple alignments of these sequences were carried out by ClustalW 1.83 version of EBI^2^ with 0.5 transition weight. The 16S rRNA sequences of the bacteria was submitted to NCBI Genbank for obtaining the accession number.

### Screening for Biocontrol Activity

#### Chitinase Test

Cultures of bacterial isolates were spot inoculated on colloidal chitin agar medium and kept in an incubator at 30°C for 5 days to check the activity of chitinase. The test was marked as positive if there was a zone of clearance around the colony (Sharma et al., 2020).

#### Cellulase Test

The culture was streak inoculated on cellulose agar containing carboxymethyl cellulose (CMC) or cellulose as sole carbon source at pH 7.0 and incubated at 30°C for 48 h. The isolate showing growth on cellulose agar was considered cellulase positive. After incubation, plates having colonies were repeatedly treated with 1% Congo Red with intermittent washing by 1 M NaCl solution. Cellulase producers developed a clear zone around the colony (Apun et al., 2000).

#### Extraction of Bioactive Compounds and Antimicrobial Screening

To extract the different bioactive compounds, the isolated bacterial cultures were first inoculated into minimal media broth and incubated at 37°C for 5 days on orbital shaker. The inoculum media comprised of (g/L) 20 g glucose, 10 g peptone, 0.5 g NaCl, 2 g CaCO_3_, 2 g KNO_3_, 0.8 g K_2_HPO_4_, 0.7 g MgSO_4_.7H_2_O, 0.5 g KCl, 1 g yeast, 0.002 g MnCl_2_, and 0.002 g FeSO_4_ and was adjusted at pH 7. The bacterial cells were then separated from the liquid medium by using a refrigerated centrifuge at 12,000 *g* for 10 min. The light brown colored supernatant was used to extract the bioactive compounds in 350 ml of different organic solvents, *viz* methanol, ethanol, ethyl acetate, and acetone each by liquid-liquid extraction method, and the extract was concentrated using a rotary evaporator. The bioactive components present in the different organic crude extracts were then tested for their antibacterial activity by Kirby–Bauer agar well diffusion assay against diverse bacteria and by dual culture plate assay for fungi, which were previously available in our lab. The microbes selected to determine the antimicrobial activity of the crude extracts of *Burkholderia seminalis* JRBHU6 were Gram-negative bacteria (*Pseudomonas aeruginosa*, *Escherichia coli* DH5α, *Klebsiella pneumonia*, and *Shigella boydii*), Gram-positive bacteria (*Staphylococcus aureus*) and fungi, *viz Fusarium oxysporum*, *Aspergillus niger*, *Microsporum gypseum*, *Trichophyton mentagrophytes*, and *Trichoderma harzianum*. Nutrient broth and nutrient agar media were used throughout the experiments for bacteria. For fungus, Sabouraud Dextrose broth and Sabouraud Dextrose agar (SDA) were used during the experiments. Amphotericin B for fungus and streptomycin for bacteria were used as positive control. Stock solutions were prepared by dissolving 100 mg of dried crude extract in dimethyl sulfoxide (DMSO). From these stock solutions, 50 μg/ml of working concentration was prepared. Thirty microliters (10^9^ CFU ml^–1^) of test microorganism was spread on nutrient agar plates. Four wells (8 mm in diameter) were cut into the agar media with a sterilized cork borer; 50 μl of crude extract of each solvent containing 50 μg/ml was poured into the respective well. Fifty microliters of antibiotic (50 μg/ml) and DMSO (50 μl per well) were also poured into one well each per plate as positive and negative control, respectively. Inoculated plates were then incubated in biological oxygen demand (BOD) incubator at 28°C for 24 h, and the zone of inhibition was measured in millimeters. The effect of methanolic crude extract on cell viability was further confirmed by epifluorescence microscopy (Nikon INTENSILIGHT C- HGFI, JAPAN) following Tawakoli et al. (2013) in different pathogenic bacteria. The number of cells was observed in randomized microscopic ocular grid fields (0.0156 cm^2^ grid area) at 40x magnification. Two different light filters were used for discriminating the dead and living cells (TRICT filter for the visualization of dead cells: excitation 528–553 nm, emission 590–650 nm; FITC filter for live cells: Excitation 465–495 nm, emission 515–555 nm).

#### Statistical Analysis

All the antimicrobial activities were performed thrice and the values represented are mean of three replications ± standard error (SE). The data was subjected to analysis of variance (ANOVA) using SPSS Ver. 16 (SPSS Inc., Chicago, IL). The treatment mean values were compared with Duncan’s multiple range tests at *P* ≤ 0.05 significance level.

### Characterization of Antimicrobial Compounds

Maximum antimicrobial activity was observed in methanolic crude extract when compared to other organic solvents, and therefore the bioactive components present in the methanolic crude extract was subjected to further purification and characterization through the following methods.

#### Thin Layer Chromatography

The methanolic crude extract was fractionated on silica gel thin layer chromatography (TLC) plates. Different mobile phases were prepared separately using mixtures of solvents, methanol, and water (2:1 v/v) used as standard mobile phase. Methanolic crude extract was applied as a spot on TLC plate and left for complete separation using the previously prepared mobile phases. The plates stood for solvent evaporation, and the R_f_ value of spots was recorded using ordinary and UV lamps, respectively.

#### Reverse-Phase High-Performance Liquid Chromatography

High-performance liquid chromatography (HPLC), a very popular and widely used method to separate, identify, and quantify compounds from a mixture, was used to identify the antimicrobial compounds in the bacterial extract. The compounds separated through TLC were curettaged and was subjected to reverse-phase HPLC (RP-HPLC) (Rao et al., 2017). The purity of the antimicrobial compounds was analyzed by analytical HPLC supported with Waters Spherisorb 5 μm ODS2 4.6 mm × 250 mm analytical cartridge (C-18 column) on a Waters 515 pump, isocratic reverse phase system with a 2,998 photodiode array detector at 210 nm, and the range given was 190–600 nm (Elshafie et al., 2017a, c). The flowrate was 1.0 ml/min, and additional UV detector was measured at 254 nm using Empower 2 software. Methanol and water (2:1) were used as the mobile phase. The purified bacterial methanol extract was mixed with HPLC-grade methanol and filtered by using a 0.22-micromillipore membrane filter before injecting into the injection port. The samples were run for 15 min, and the retention time was noted; based on the percentage of the peak area, the purity of each compound was determined.

#### Gas Chromatography–Mass Spectrometry Analysis

Gas chromatography–mass spectrometry (GC/MS) analysis was conducted as explained by Gohar et al. (2010) with slight differences in analytical conditions. HPLC-eluted compounds were analyzed using Thermo Scientific gas chromatography coupled with ITQ 1100 mass detector and X-Caliber software and National Institute of Standards and Technology (NIST) spectral data (GCMS, Thermo Fisher Scientific) with DB-5 MS capillary column (30 μm × 0.25 μm internal diameter and 0.25 μm film thickness). Analytical chromatographic conditions were as follows: 2 μl sample injected; carrier gas helium at 1 ml/min; injector and transfer line temperatures used were 220 and 280°C, respectively; over temperature program included 50°C–2 min hold, 150°C/min rate; increased to reach 160°C and remained at temperature for 0 min hold, 50°C/min rate; increased to reach 200°C and remained at temperature for 1 min hold, 2°C/min rate; increased to reach 230°C and remained at temperature for 1 min hold, 80°C/min rate; increased to reach 285°C and remained at temperature for 6 min hold; split ratio = 1:50; ionization energy, 70eV; mass range, 50–650. The composition was determined by comparing peak retention times with those of reference standards.

#### Fourier-Transform Infrared Spectroscopy Analysis

Using JASCO Fourier-transform infrared (FTIR) spectrometer, the molecular structure of the compound was partially identified. HPLC eluted compounds were dissolved in dry KBr, which was later subjected to FTIR spectroscopy. The measurement was carried out at infrared spectra in the region 600–4,000 nm cm^–1^, using ATR accessories.

#### Nuclear Magnetic Resonance Spectroscopy Analysis

^1^H NMR spectra were acquired by dissolving the purified compound eluted from HPLC in deuterated (d_6_) DMSO solvent at a concentration of 1 mg ml^–1^ and analyzed on a JEOL-500 MHz NMR spectrometer.

### *In silico* Target Identification

The antimicrobial activity of the bioactive compounds was elucidated through phenotypic bacterial and fungal growth assay. Since it is unclear whether such phenotypic observations resulted from general cytotoxicity or they were target specific, an *in silico* approach was made to identify the targets of these compounds. The targets were predicted using structural similarity and pharmacophore-based approaches.

#### Target Prediction Based on the Chemical Structure

This target prediction method is based on the principle that the similar chemical structures tend to perform similar biological functions (Willett et al., 1998; Bender and Glen, 2004). Thus, the chemical structures that share structural or substructural similarity will interact with similar protein target.

#### Target Prediction Based on Pharmacophore

PharmMapper^3^ (Wang et al., 2017) web service was used for pharmacophore-based target predictions. In order to derive a dataset, pharmacophores derive structures complexed with small molecules from Binding DB, DrugBank, PDB Bind, and the PDTD databases. Results are discussed in “*In silico Target Identification*.”

## Results

### Isolation, Molecular Identification, and Phylogenetic Analysis of Bacterial Isolate

From the composite soil samples collected from different drought-exposed areas, a total of 53 bacterial isolates were obtained and screened for their morphological, biochemical, and biocontrol potentials. The most potent bacterial isolate selected was JRBHU6, which had moist, raised, yellow colored, undulated margin colonies and was Gram negative, rod shaped, and positive for Voges--Proskauer, mannitol, and catalase test and negative for methyl red test. Taxonomic affiliation of the isolate JRBHU6 based on 16S rRNA sequencing revealed the isolate as *B. seminalis.* A comparison of 16S rRNA gene sequences with those of *B. seminalis* strains available at NCBI revealed similarity levels above 99%. The 16S rRNA sequences of *B. seminalis* JRBHU6 was submitted to NCBI, GenBank under the accession number MK500868. Phylogenetic trees were constructed in MEGA 10.0 version^4^ using a maximum parsimony algorithm (Supplementary Figure 1). Interestingly, *B. seminalis* JRBHU6 formed a separate branch in the phylogenetic tree and hence was marked to be novel.

### Biocontrol and Antimicrobial Activity

#### Chitinase Test

**FIGURE 1:**
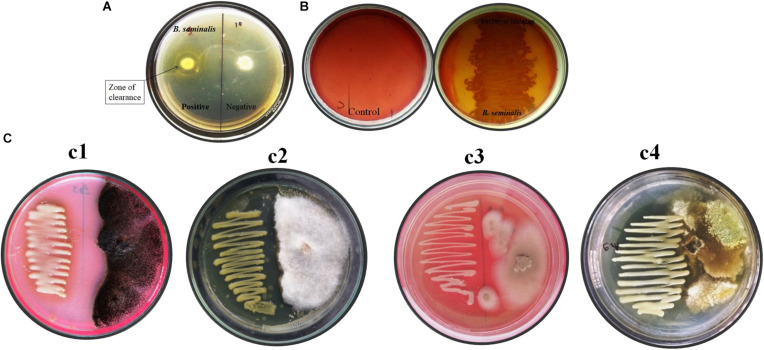
*In vitro* assay of enzymatic and antifungal activity exhibited by *B. seminalis* JRBHU6. **(A)** Chitinase activity **(B)** Cellulase activity **(C)** Antifungal activity against different fungus (Cl-*Aspergillus niger*, C2*-Fusarium oxysporum-*, C3*-Microsporum gypseum, and* C4-*Trichoderma harzianum*).

*Burkholderia seminalis* JRBHU6 showed a clear halo zone around colony-confirming solubilization of colloidal chitin in nutrients agar media supplemented with 1% colloidal chitin after incubation at 30°C for 5 days (Figure 1A).

#### Cellulase Test

*Burkholderia seminalis* JRBHU6 showed a zone of clearance around colony indicating solubilization of carboxymethylcellulose (CMC) in nutrients agar media, confirming its cellulose-degrading ability (Figure 1B).

#### Antifungal Activity

*Burkholderia seminalis* JRBHU6 showed strong antifungal activity against all the fungal strains, *viz F. oxysporum*, *A. niger*, *M. gypseum*, *T. mentagrophytes*, and *T. harzianum* incubated at 25°C for 7 days (Figure 1C). The high inhibitory activity of *B. seminalis* JRBHU6 against all the selected soil fungi indicated the secretion of antifungal compounds into the agar medium in dual culture plate assay which restricted all the selected fungi to less than 50% area of the plate. The antagonistic behavior of *B. seminalis* JRBHU6 was evident from the suppressed mycelial growth along with the arrested spore/sclera formation in the fungi.

#### Antibacterial Activity

**FIGURE 2:**
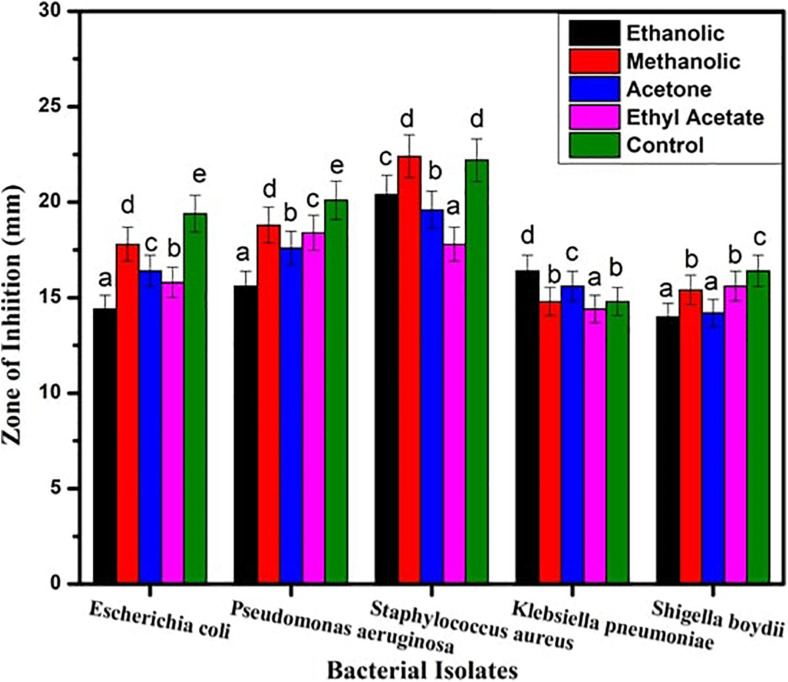
Antimicrobial activity exhibited by crude extract *of B. seminalis* JRBHU6 obtained in different organic solvents against various bacteria. Results are expressed as means of the replicates with the vertical bars indicating the error of the means. Different letters indicate significant differences among treatments results according to Duncan’s multiple range test at *P* < 0.05.

The inhibitory action of *B. seminalis* JRBHU6 crude extract obtained in different organic solvents was observed in terms of diameter of the inhibition zone formed around the agar well by diffusion of antimicrobial substances following Kirby–Bauer agar well diffusion method. The zone of clearance around the well indicated the positive antibacterial activity given by the test sample. Maximum inhibition zone formed by the derived crude extracts of *B. seminalis* JRBHU6 was observed in methanolic crude extract tested against *S. aureus*, which was 22.4 mm and in ethanolic crude extract (20.4 mm). The methanolic crude extract proved better than other organic extract in exhibiting a broad spectrum inhibition activity against the gram-negative *P. aeruginosa* (18.8 mm), *E. coli* DH5α (17.8 mm), *S. boydii* (15.4 mm), and *K. pneumonia* (14.8 mm) strains. The diameter of the inhibition zone formed by the methanolic crude extract against each microorganism was found to be equal or greater than that of the standard antibiotic streptomycin (22.2 mm) used in the assay (Figure 2). The different bacterial pathogenic cultures exposed to 18 hrs in methanolic crude extract of *B. seminalis* JRBHU6 were also examined by epifluorescence microscopy to check their viability. Epifluorescent microscopic images clearly confirmed the bactericidal nature of the crude extract (Supplementary Figure 2).

### Characterization of Compounds

The bioactive compounds present in methanolic crude extract were identified and characterized by using TLC, HPLC, GC-MS, FTIR, and NMR spectroscopic techniques.

#### Thin Layer Chromatography

Separation of compounds present in *B. seminalis* JRBHU6 methanolic crude extract was best observed in methanol and water (2:1 v/v) solvent system as compared to other tested solvent systems. A single brown spot was separated. The Rf values for the colored spot was 0.71.

#### HPLC

**FIGURE 3:**
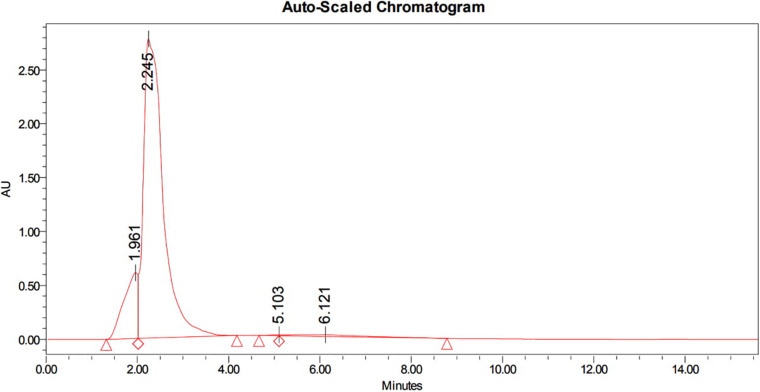
HPLC chromatogram of methanolic crude extract of *B. seminalis* JRBHU6 obtained at PDA 210 nm showing characteristic peaks with retention time 1.961 min., 2.245 min., 5.103 min. and 6.121 min. The eluted active compounds at retention time 2.245 min. having the highest peak area was subjected to further characterization studies.

The bioactive compounds in methanolic extract was purified by HPLC, which showed a characteristic peaks with retention time 1.961 min, 2.245 min, 5.103 min, and 6.121 min at PDA 210 nm (Figure 3). The eluted active compounds at retention time 2.245 min having the highest peak area was subjected to further characterization by GC-MS, FTIR, and ^1^H-NMR studies.

#### GC-MS Analysis

**FIGURE 4:**
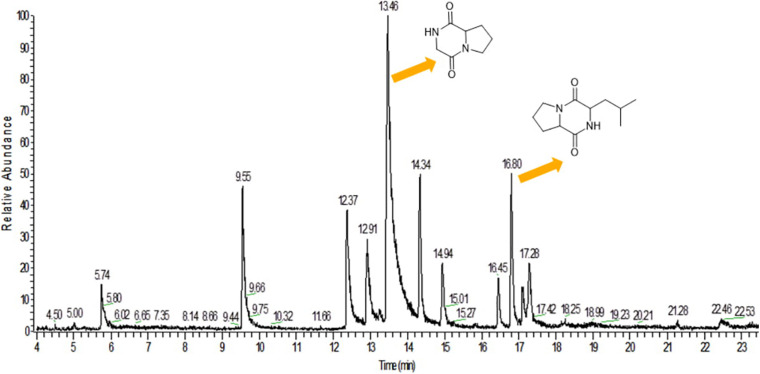
Gas Chromatography analysis of methanolic crude extract of *B. seminalis* JRBHU6. Chemical structures of identified compounds are indicated against peaks.

**FIGURE 5:**
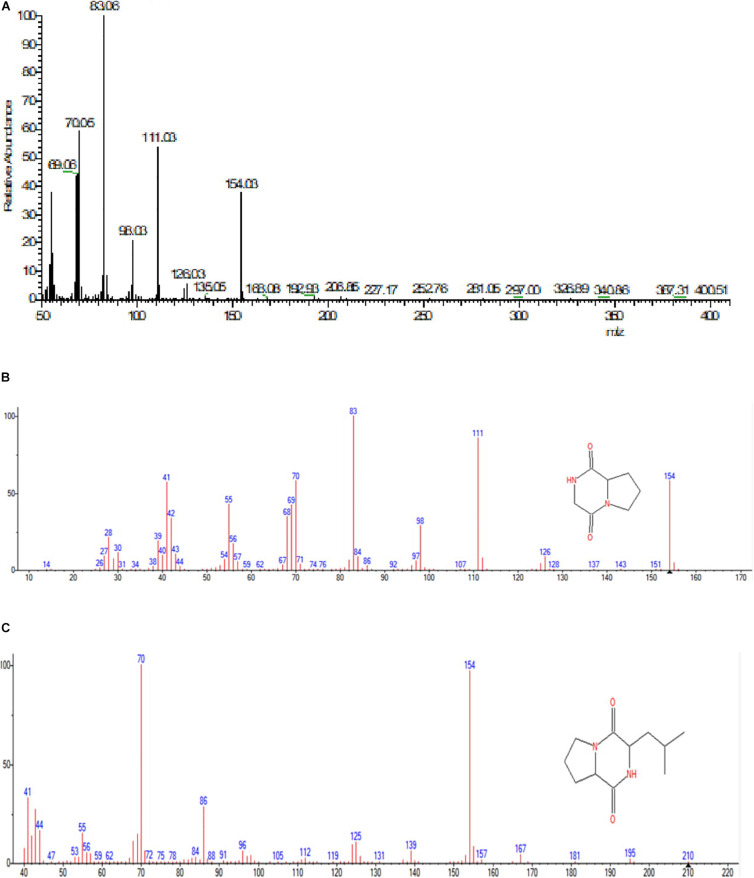
**(A)** Mass spectral analysis of methanolic crude extract of *B. seminalis* JRBHU6. **(B)** Full range spectra of PPDH compound. **(C)** Full length spectra of PPDHMP compound.

GC-MS analysis was performed to identify the bioactive molecules present in the methanolic extract. GC-MS analysis showed the presence of multiple compounds in the sample based on molecular weight, retention time, and molecular formula when compared with the NIST library. The peak area corresponds to the quantity of the compound present in the sample. Based on GC-MS spectra (as depicted in Figures 4, 5), the two most intense peaks at 13.46 min corresponding to PPDH present in the sample showing mass of 154 Da with molecular formula C_7_H_10_O_2_N_2_ [pyrrolo(1,2-a)pyrazine-1,4-dione,hexahydro] and at 16.80 min corresponding to PPDHMP present in the sample analyzed showing mass of 210 Da with molecular formula C_11_H_18_N_2_O_2_ [pyrrolo (1,2-a)pyrazine-1,4-dione, hexahydro-3(2-methylpropyl)] were identified.

#### FTIR Spectrum

**FIGURE 6:**
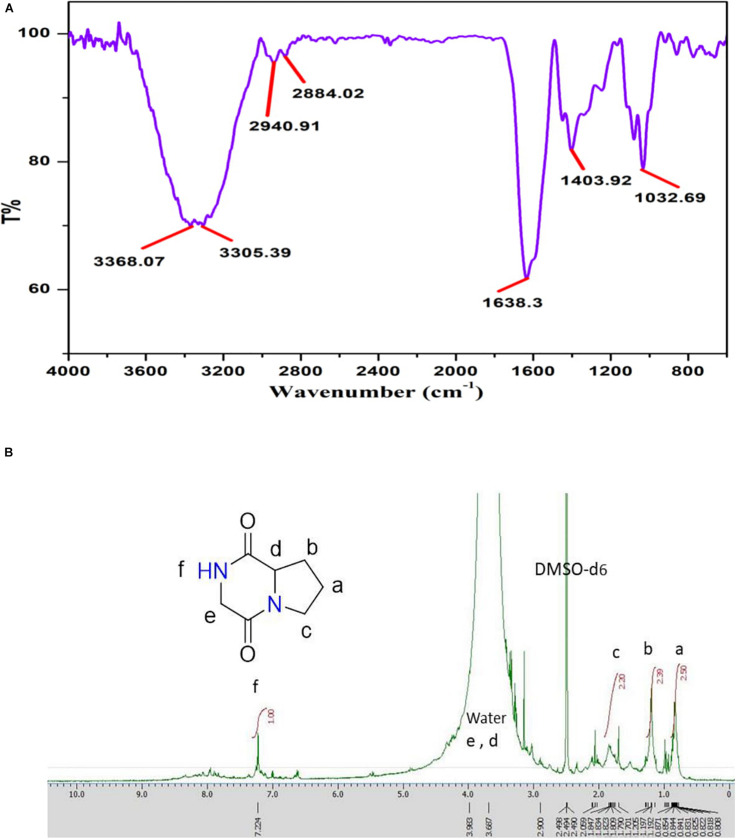
**(A)** FT-IR spectrum showing stretching frequency of functional groups of compound PPDH. **(B)** ^1^H-NMR spectrum of PPDH.

The major peak obtained for Pyrrolo[1,2-a]pyrazine-1,4-dione,hexahydro (PPDH) was further studied through FTIR spectroscopy. FTIR spectrum of PPDH showed the characteristic peaks at 3,400–3,140 cm^–1^ corresponding to amide N–H group, 2,940 cm^–1^ corresponding to alkyl C–H group, and at 1,638 cm^–1^ indicating C = O stretching of amide, respectively (Figure 6A).

#### ^1^H-NMR

The ^1^H-NMR spectrum (JEOL 500 MHz, solvent; DMSO-d_6_) showed signals at δ = 0.8 (2H, q), 1.205 (2H, m), 1.809 (2H, t), 3.108 (2H, s), and at 3.411(1H, t) ppm corresponding to aliphatic protons and peak at 7.236 ppm corresponding to N–H proton (Figure 6B).

The isolated bioactive PPDH fully characterized by GC-MS, HPLC, FTIR, and NMR techniques was confirmed as pyrrolo(1,2-a)pyrazine-1,4-dione,hexahydro (Figure 6B).

### *In silico* Target Identification

PPDH and PPDHMP, the two bioactive compounds characterized by GC-MS present in the crude extract of *B. seminalis* JRBHU6, were selected for further studies. Their inhibitory activity through methanolic crude extract was already elucidated through *in vitro* bacterial and fungal growth assay. An *in silico* approach was further used to identify the potential targets against the identified compounds. Since the enzyme Chitinase B protein (PDB ID: 1W1P) is a well-known target of PPDH and is well reported in Protein Data Bank (PDB), therefore, targets for only PPDHMP were predicted using structure and pharmacophore-based approaches.

#### Target Prediction Based on Structure of PPDHMP

The target prediction method was based on the principle that the similar chemical structures tend to perform similar biological functions (Willett et al., 1998; Bender and Glen, 2004) and therefore will interact with similar protein target. Keeping this in view, the smile structure of PPDHMP [(CC(C)CC1C(= O)N2CCCC2C(= O)N1)] was submitted to PubChem database (with similarity ≥ 70%) for chemical similarity search. Results showed the chemical similarity with our studied PPDH. In this regard, chitinase B could also be the likely target for PPDHMP.

#### Target Prediction Based on Pharmacophore

All the predicted targets along with their functions are given in Table 1. Interestingly, all the identified targets were enzymes. Results were analyzed based on their Z-score value (Table 1). A large positive Z-score indicates high significance of the target to a query compound. All predicted targets with a Z-score > 1.0 were considered likely targets. Interestingly, 1W1P was one of the predicted targets along with human and bacterial proteins (Table 1). All likely targets were further subjected to docking analysis.

**TABLE 1:** Predicted targets for compound PPDHMP identified in *B. seminalis* JRBHU6.

PDB ID	Organism	Protein	Functions	Z-score
1l2s	*E. coli*	Beta-lactamase	Substrate specificity for cephalosporins	3.84
1iow	*E. coli*	D-alanine-D-alanine ligase B	Cell wall formation	3.72
1ua2	*Homo sapiens*	Cell division protein kinase 7	Involved in protein kinase activity	3.58
1gyy	*E. coli*	Probable tautomerase ydcE	Signal transduction mechanisms	3.08
2bjf	*C. perfringens^a^*	Choloylglycine hydrolase	Cell wall/membrane/envelope biogenesis	2.11
1uki	Human	Mitogen-activated protein kinase 8	Responds to activation by environmental stress and pro- inflammatory cytokines	1.60
1ez2	*B. diminuta^b^*	Parathion hydrolase	Substrate specificity for synthetic organophosphate triesters and phosphorofluoridates	1.53
1ci9	*B. gladioli^c^*	Esterase EstB	Involved in beta-lactamase activity	1.46
1naq	*E. coli*	Divalent-cation tolerance protein cut A	Inorganic ion transport and metabolism	1.45
1ukb	*P. simple^d^*	2-hydroxy-6-oxo-7-methylocta-2,4-dienoate hydrolase	Involved in hydrolase activity	1.40
1rf9	*P. putida^e^*	Camphor 5-monooxygenase	Secondary metabolites biosynthesis, transport and Catabolism	1.30
1w1p	*S. marcescens^f^*	chitinase B	–	1.26
1srj	*S. avidinii^g^*	Streptavidin	Forms a strong non-covalent specific complex with biotin	1.22
1qpn	*M. tuberculosis* H37Rv*^h^*	Nicotinate-nucleotide pyrophosphorylase (carboxylating)	Coenzyme transport and metabolism	1.21
1sup	*B. amyloliquefaciens^i^*	Subtilisin BPN	Post-translational modification, protein turnover, Chaperones	1.10
2tpl	*C. freundii^j^*	Tyrosine phenol-lyase	Amino acid transport and metabolism	1.04

*Clostridium perfringens^a^, Brevundimonas diminuta^b^, Burkholderia gladioli^c^, Pimelobacter simplex^d^, Pseudomonas putida^e^, Serratia marcescens^f^, Streptomyces avidinii^g^, Mycobacterium tuberculosis H37Rv^h^, Bacillus amyloliquefaciens^i^, and Citrobacter freundii ^j^.*

#### Docking Analysis of Compounds

Ligand-based docking technique was implemented in this work to study the binding interactions between identified bioactive compounds PPDH and PPDHMP and their protein targets. All molecular docking was carried out using SwissDock web service. Results were analyzed based on binding free energy (ΔG) and full fitness of compounds to their targets. The ΔG and full fitness of PPDH was calculated as −6.43 and −1,584.6 kcal/mol, respectively. The values of ΔG and full fitness for PPDHMP are given in Table 2. The energy above the threshold values of ΔG and full fitness ≥ −6.42 and −1,583.48 kcal/mol, respectively, for PPDHMP was considered for further analysis. Interestingly, PPDHMP has shown strong full fitness to human proteins: cell division protein kinase7 (−1,561.97 kcal/mol) and mitogen-activated protein kinase 8 (−1,964.97 kcal/mol) with significant ΔG values of −6.81 kcal/mol. Besides human proteins, it has also shown good binding affinity and full fitness with bacterial proteins: choloylglycine hydrolase, camphor 5-monooxygenase, chitinase B, and tyrosine phenol-lyase.

**TABLE 2:** Docking analysis of PPDH and PPDHMP with its likely targets.

Organism	Protein	ΔG (kcal/mol)	Fullfitness (kcal/mol)
	**PPDH**		
*Serratia marcescens*	Chitinase B (1W1P)	−6.42	−1,583.48
	**PPDHMP**		
*Escherichia coli*	Beta-lactamase (1L2S)	−6.58	−1,369.07
*Escherichia coli*	D-alanine–D-alanine ligase B (1IOW)	−6.25	−1,373.69
*Homo sapiens*	Cell division protein kinase 7 (1UA2)	−6.81	−1,561.97
*Escherichia coli*	Tautomerase ydcE (1GYY)	−6.58	−491.25
*Clostridium perfringens*	Choloylglycine hydrolase (2BJF)	−7.10	−1,643.38
*Homo sapiens*	Mitogen-activated protein kinase 8 (1UKI)	−6.81	−1,964.97
*Brevundimonas diminuta*	Parathion hydrolase (1EZ2)	−6.40	−1,219.33
*Burkholderia gladioli*	Esterase EstB (1CI9)	−6.84	−1,171.30
*Escherichia coli*	Divalent-cation tolerance protein cut A (1NAQ)	−6.76	−562.42
*Pimelobacter simplex*	2-hydroxy-6-oxo-7-methylocta-2,4-dienoate hydrolase (1UKB)	−7.21	−1,261.47
*Pseudomonas putida*	Camphor 5-monooxygenase (1RF9)	−6.98	−1,699.01
*Serratia marcescens*	Chitinase B (1W1P)	−6.42	−1,583.48
*Streptomyces avidinii*	Streptavidin (1SRJ)	−7.17	−475.12
*Mycobacterium tuberculosis* H37Rv	Nicotinate-nucleotide pyrophosphorylase (carboxylating) (1QPN)	−6.26	−1,287.85
*Bacillus amyloliquefaciens*	Subtilisin BPN (1SUP)	−6.47	−631.26
*Citrobacter freundii*	Tyrosine phenol-lyase (2TPL)	−6.72	−2,074.66

**FIGURE 7:**
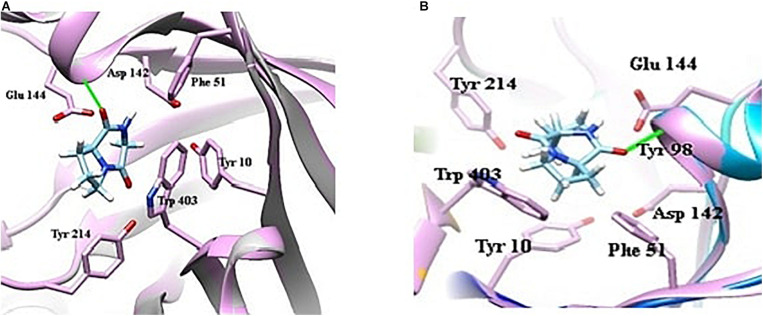
Docking view of bacterial protein chitinase B *(Serrattia marcescens)* with compounds identified in *B. seminalis* JRBHU6. **(A)** Represents the docking of PPDH. **(B)** Represents the docking of PPDHMP.

**FIGURE 8:**
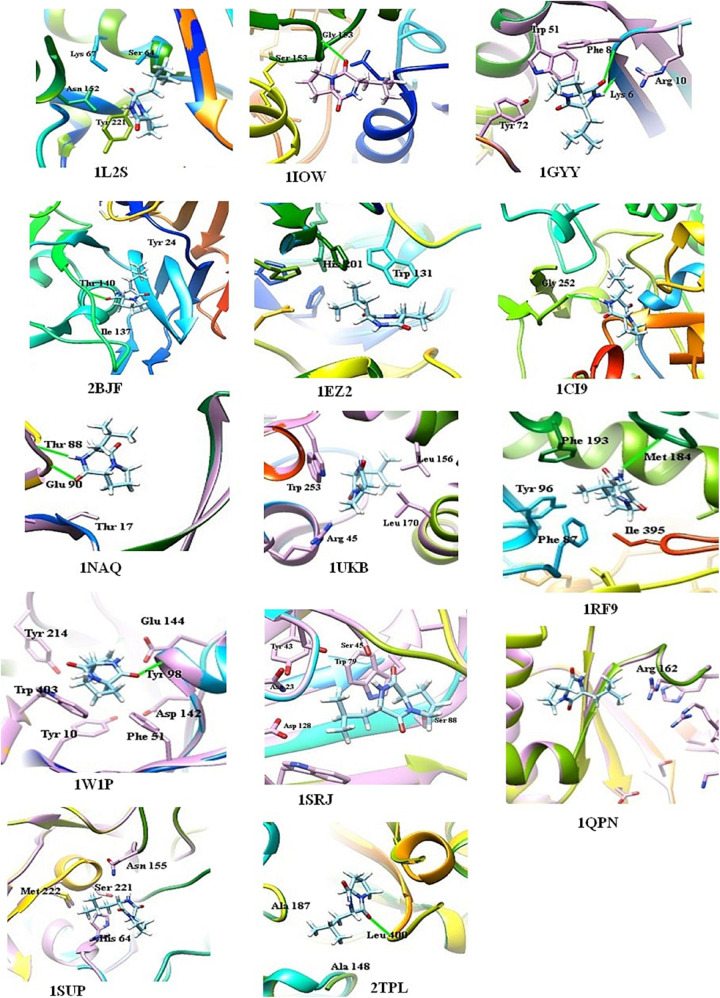
Binding interactions of PPDHMP compound identified in *B, seminalis* JRBHU6 with predicted targets.

Docking of chitinase B protein with both PPDH and PPDHMP was observed. The PPDHMP has also shown almost the same binding free energy (−6.42 kcal/mol) and full fitness (−1,583.48 kcal/mol) with chitinase B (Table 2). Residues of chitinase B protein, *viz* Tyr10, Phe51, Asp142, Glu144, Tyr214, and Trp403, were found to be actively involved in binding interaction with both PPDH and PPDHMP (Figures 7A,B). Residue Asp142 of chitinase B made an H-bond interaction with the PPDH (Figure 7A), while residue Tyr98 made an H-bond interaction with PPDHMP (Figure 7B). Docking results of PPDHMP with its other targets are enumerated in Figure 8 and Table 2. While PPDHMP showed good binding energy with all its targets, it showed poor full fitness toward tautomerase (−491.25 kcal/mol), divalent-cation tolerance protein (−562.42 kcal/mol), streptavidin (−475.12 kcal/mol), and subtilisin BPN proteins (−631.26 kcal/mol). However, it showed good binding affinity and full fitness with bacterial proteins: choloylglycine hydrolase, camphor 5-monooxygenase, and tyrosine phenol-lyase.

Docking results indicate that both compounds have shown good binding affinity toward their targets. Strong binding of these compounds with proteins and enzymes may inhibit the enzymatic activities in the pathogen leading to growth inhibition, which can be the reasons for the observed antimicrobial effects. The observed *in silico* studies, however, needs experimental validation to decipher the exact mechanism involved in growth inhibition. Interestingly, PPDHMP has also shown strong binding affinity to some of human proteins: cell division protein kinase 7 (−1,561.97 kcal/mol) and mitogen-activated protein kinase 8 (−1,964.97 kcal/mol) with significant ΔG values of −6.81 kcal/mol. Molecular docking results completely support the multitargeting nature of PPDHMP. Our *in silico* docking results provide a strong platform to extend the studies further in this direction.

## Discussion

Antimicrobial agents are low molecular weight organic compounds having diverse groups that are synthesized by microbes, which, even at low concentrations, inhibit the growth or metabolic activity of other microbes (Duffy et al., 2003). There is growing awareness on development of new antimicrobial drugs, inspired by natural resources that can significantly contribute in the treatment of human, animal, and plant disease. Soil bacteria are a rich source of antimicrobial compounds like peptides, terpenoids, polyketides, polysaccharides, fatty acids, alkaloids, steroids, and phenolic compounds. In today’s date, almost 90% of clinically used antibiotics are from microbial sources (Katz and Baltz, 2016) with majority of them discovered from soil bacteria through antimicrobial screening (Rolain et al., 2016). The Gram-negative bacteria *Burkholderia* selected in the present study are rod-shaped obligate aerobes, having diverse ecological niches (Compant et al., 2008). These niches range from soil and plant reservoirs to mammalian host reservoirs (Coenye and Vandamme, 2003). Presently, it was isolated from rhizospheric soil where the dwelling microorganisms may act as biocontrol agents to protect plants roots against pathogens (Weller, 1988; Raaijmakers et al., 2002). The antagonistic activity of *Burkholderia* spp. predominantly against phytopathogenic fungi and oomycetes is well recognized (Hebbar et al., 1992), and several secondary metabolites have been tested by agar well diffusion and characterized by GC-MS method. A potent species *Burkholderia phytofirmans* has the ability to colonize the rhizosphere of majority of plants and enhance plant growth (Sessitsch et al., 2005). *Burkholderia* species also has the potential to enhance plant nutrient availability *via* phosphate solubilization and/or nitrogen fixation, e.g., *B. tropica* and *B. unamae* (Reis et al., 2004), and others, like *B. uname*, have a potential to catalyze aromatic compounds (Caballero-Mellado et al., 2007). *Burkholderia* spp. has been well documented for its immense potential in disease suppression through antimicrobial metabolite production (Perin et al., 2006; Vial et al., 2007; Araújo et al., 2016) and production of antibiotics and siderophores (Vial et al., 2011; Ong et al., 2016). However, the roles of siderophores that are mainly creating a competitive environment in the rhizosphere and the other antagonistic mechanisms exerted by this genus have been poorly understood, except for the clinical isolates *B. cepacia* and *B. vietnamensis*, for which cepabactin (siderophores of the hydroxamate class), salicylic acid (catechol type), ornibactin, and pyochelin have been recognized (Meyer et al., 1995). Araújo et al. (2017), in an interesting study, evaluated the inhibitory activity of *B. seminalis* TC3.4.2 R3 by coculturing it with *F. oxysporum* and, through matrix-assisted laser desorption/ionization mass spectrometry imaging (MALDI-MSI), identified the secondary metabolites pyochelin and rhamnolipid, which work in synergy with other unidentified molecules in inhibiting the growth of the pathogen. An umbrella of secondary metabolites produced by the genus *Burkholderia* possesses antifungal (Lu et al., 2009), antibacterial (Mitchell et al., 2008), and antitumor (He et al., 2014) properties against exotoxins (Partida-Martinez and Hertweck, 2007) activity. Numerous metabolites with antifungal activities, comprising cepacins, cepaciamides, cepacidines, siderophores, quinolones, phenazines, pyrrolnitrins, and lipopeptides (Huang and Wong, 1998), have been reported for *Burkholderia* species (Mao et al., 2006). Most of the antibiotics isolated from *Burkholderia* culture filtrates belong to the class of N-containing heterocycles like pyrrolnitrin-type antibiotics, phenazines, pyoluteorin, and indole derivatives and originate as intermediates or end products during the aromatic amino acid biosynthesis (Leisinger and Margraff, 1979).

Application of antagonistic microorganisms is an eco friendly approach to effectively control diseases in plants (Yuan and Crawford, 1995). The taxonomic position of strain *B. seminalis* JRBHU6 was worked out using polyphasic approach. The16S rRNA sequence of strain *B. seminalis* JRBHU6 revealed the genus *Burkholderia* with close similarity to *B. seminalis*. Phylogenetic analysis further confirmed the strain JRBHU6 to clade closely with *B. seminalis*. In spite of showing high 16S rRNA gene sequence similarity, phylogenetic analysis indicated the strain JRBHU6 to be positioned on a different branch of the phylogenetic tree, signifying it as a novel strain. Strains of *Burkholderia* are well-known for showing high similarity in sequences of 16S rRNA gene (Peeters et al., 2013). In the present study, *B. seminalis* JRBHU6 isolated from drought-exposed field exhibited strong antimicrobial activities. The biocontrol efficiency of any microorganism is credited to the array of hydrolytic enzymes and bioactive compounds mediating antimicrobial activity (Caulier et al., 2019). Among the hydrolytic enzymes, cellulase and chitinase are worth mentioning, as they target chitin, which is one of the major constituents of fungal cell wall (Gomaa, 2012; Geraldine et al., 2013), and the literature remarkably addresses many bacterial strains with antimicrobial activity. *B. seminalis* JRBHU6 also exhibited strong chitinase and cellulase activity, which helps in imposing its biocontrol activity. Chitinases have been earlier reported and characterized mainly in the strain of *B. gladioli* (Kong et al., 2001; Ogawa et al., 2002). The literature on cellulase production by different *B. altitudinis* strains (Sreeja et al., 2013; Verma et al., 2015) is well documented. However, there are fewer reports on the production of more than one hydrolytic enzyme by *B. seminalis.* Interestingly, *B. seminalis* JRBHU6 was positive for both chitinase as well as cellulase activity. Elshafie et al. (2017c), through GC-MS analysis, identified methyl stearate and ethanol 2-butoxy phosphate in *Burkholderia gladioli* pv *agaricicola*, which, together with extracellular hydrolytic enzymes, exhibited antimicrobial affects. A related study done by Mehdi et al. (2006) revealed *Streptomyces* sp. TN97 to produce isocoumarin derivative, diketopiperazine derivatives, and the *N*-acetyltyramine exhibiting potent antimicrobial activities. Screening crude extract in different solvents to get the maximum number and the highest amount of bioactive compounds is crucial to begin with. The idea is well supported by Kobayashi et al. (1994) and Khamna et al. (2009), who revealed that ethyl acetate extract displayed high antimicrobial activity against fungi and bacteria. In the present work, too, crude extract of *B. seminalis* JRBHU6 was purified in different solvents, *viz* ethyl acetate, acetone, methanol, and ethanol. The maximum zone of inhibition was observed against *S. aureus* (22.4 mm) by methanolic crude extract of *B. seminalis* JRBHU6. For the physicochemical characterization of methanolic crude extract, preparative silica gel TLC was carried out on which a clear distinct spot with Rf value 0.71 was observed. In a similar study, ethyl acetate crude extract obtained from *Pseudomonads* sp. was fractionated using silica gel TLC plates, and the bioactive compound obtained at Rf value 0.75 exhibited strong antifungal activity against *Phytophthora capsici* and *Colletotrichum orbiculare* (Lee et al., 2003).

The HPLC spectrum of the methanolic crude extract showed an intense characteristic peak at the retention time of 2.245 min, which was denoted as PPDH. On the basis of spectral data, including FTIR spectroscopy, proton nuclear magnetic resonance (^1^H NMR), and mass analysis, the major isolated compounds were identified as C_7_H_10_O_2_N_2_ [pyrrolo(1,2-a)pyrazine-1,4-dione, hexahydro] (PPDH) and C_11_H_18_N_2_O_2_ [pyrrolo(1,2-a)pyrazine-1,4-dione,hexahydro-3(2-methyl-propyl)] (PPDHMP). The peaks and the functional groups revealed after FTIR were similar to the earlier reports (Mohan et al., 2016; Kiran et al., 2018). The pyrrolo(1,2-a)pyrazine group is a widely occurring natural product used in drug designing. Pyrrole has therapeutic significance as a antitumor, anti-inflammatory, and cholesterol-reducing drug (Kiran et al., 2018). It has been reported from numerous species of *Streptomyces* (Kumar and Lown, 2003; Ser et al., 2015; Sanjenbam and Kannabiran, 2016) and *Bacillus tequilensis* MS145 (Kiran et al., 2018), which are well-reported antibiotic producers. Melo et al. (2014) also screened the compounds pyrrolo(1,2-a)pyrazine-1,4-dione,hexahydro-3-(2-methyl-propyl) and pyrrolo(1,2-a)pyrazine-1,4-dione, hexahydro-3-(phenylmethyl) from *Mortierella alpina* fungus and reported their antibacterial activity against *P. aeruginosa*, *E. coli*, and *Enterococcus faecalis*.

Molecular informatics attempts to find interrelationships between molecules based on ideas and concepts derived from experimental studies. Comparative study helps in identifying the most reliable method among those that are being compared. Different similarity measures reflect the varied molecular characteristic, and with the complex nature of biological activity, no individual measure is optimal for all sorts of similarity search. Using techniques from digital image processing, Robinson et al. (1997) described the 3D structure representation of a molecule that is derived from its 2D structure in an extremely rapid process. PharmMapper, which is an open-source web server, through large-scale reverse pharmacophore mapping strategy, helps in identifying potential drug targets. Results were analyzed based on their Z-score value. High significance of the target to a query compound is reflected by a large positive Z-score. In the present study, all targets with a Z-score > 1.0 were taken as likely targets and subjected to further docking analysis. Interestingly, some of the predicted targets were human, and the rest were bacterial (Table 1). Results were also analyzed based on binding free energy (ΔG) and full fitness of the PPDHMP to the target binding site (Table 2). Among all the predicted targets, PPDHMP has shown good binding but less full fitness to tautomerase ydcE (−491.25 kcal/mol), Divalent-cation tolerance protein (−562.42 kcal/mol), streptavidin (−475.12 kcal/mol), and subtilisin (−631.26 kcal/mol) of *E. coli*, *Streptomyces avidinii*, and *Bacillus amyloliquefaciens*, respectively. However, it has shown significant full fitness to beta-lactamase and D-alanine-D-alanine ligase B proteins of *E. coli* and other bacterial proteins. Few earlier reports have shown the ability of pyrrole to inhibit the activity of reverse transcriptase of HIV-1 viruses, cellular DNA polymerases, and protein kinase activity (Bhardwaj et al., 2015; Kiran et al., 2018). Interestingly, in the present study, too, PPDHMP has shown strong full fitness to human proteins: cell division protein kinase 7 (−1,561.97 kcal/mol) and mitogen-activated protein kinase 8 (MAPK8) (−1,964.97 kcal/mol) with significant ΔG values of −6.81 kcal/mol. Results from the present study demonstrate the compounds PPDH and PPDHMP purified from *B. seminalis* JRBHU6 as potent biocontrol agents targeting many key enzymes and proteins of microbes.

## Conclusion

In the present study, *B. seminalis* JRBHU6 isolated from rhizospheric soil was found to possess strong antimicrobial activities. Spectroscopic analysis of metabolic extract revealed the bioactive metabolite as PPDH. An additional compound, PPDHMP, revealed in GC-MS on *in silico* molecular docking, showed strong full fitness while interacting with many microbial and human proteins and enzymes, which could play a significant role in microbial growth inhibition. *B. seminalis* JRBHU6 was also a strong chitinase and cellulase enzyme producer. These hydrolytic enzymes in a synergistic action with the pyrrole metabolites must be responsible for the observed antagonistic behavior of *B. seminalis* JRBHU6.

## Data Availability Statement

The datasets presented in this study can be found in online repositories. The names of the repository/repositories and accession number(s) can be found in the article/Supplementary Material.

## Author Contributions

RR and JP: conceptualization. JP, PP, and RA: methodology and investigation. RR, JP, and RA: writing—original draft preparation. RR: writing—review and editing. All authors contributed to the article and approved the submitted version.

## Conflict of Interest

The authors declare that the research was conducted in the absence of any commercial or financial relationships that could be construed as a potential conflict of interest.

## Footnotes

1 http://blast.ncbi.nlm.nih.gov/Blast.cgi

2 www.ebi.ac.uk/cgi-bin/clustalw/

3 http://www.lilab-ecust.cn/pharmmappers

4 www.megasoftware.net
