# Supplementary figures and images for: Drought Exposed Burkholderia seminalis JRBHU6 Exhibits Antimicrobial Potential Through Pyrazine-1,4-Dione Derivatives Targeting Multiple Bacterial and Fungal Proteins
Source: Front Microbiol. 2021 Apr 14;12:633036. doi: 10.3389/fmicb.2021.633036 (PMC8079638; doi:10.3389/fmicb.2021.633036)

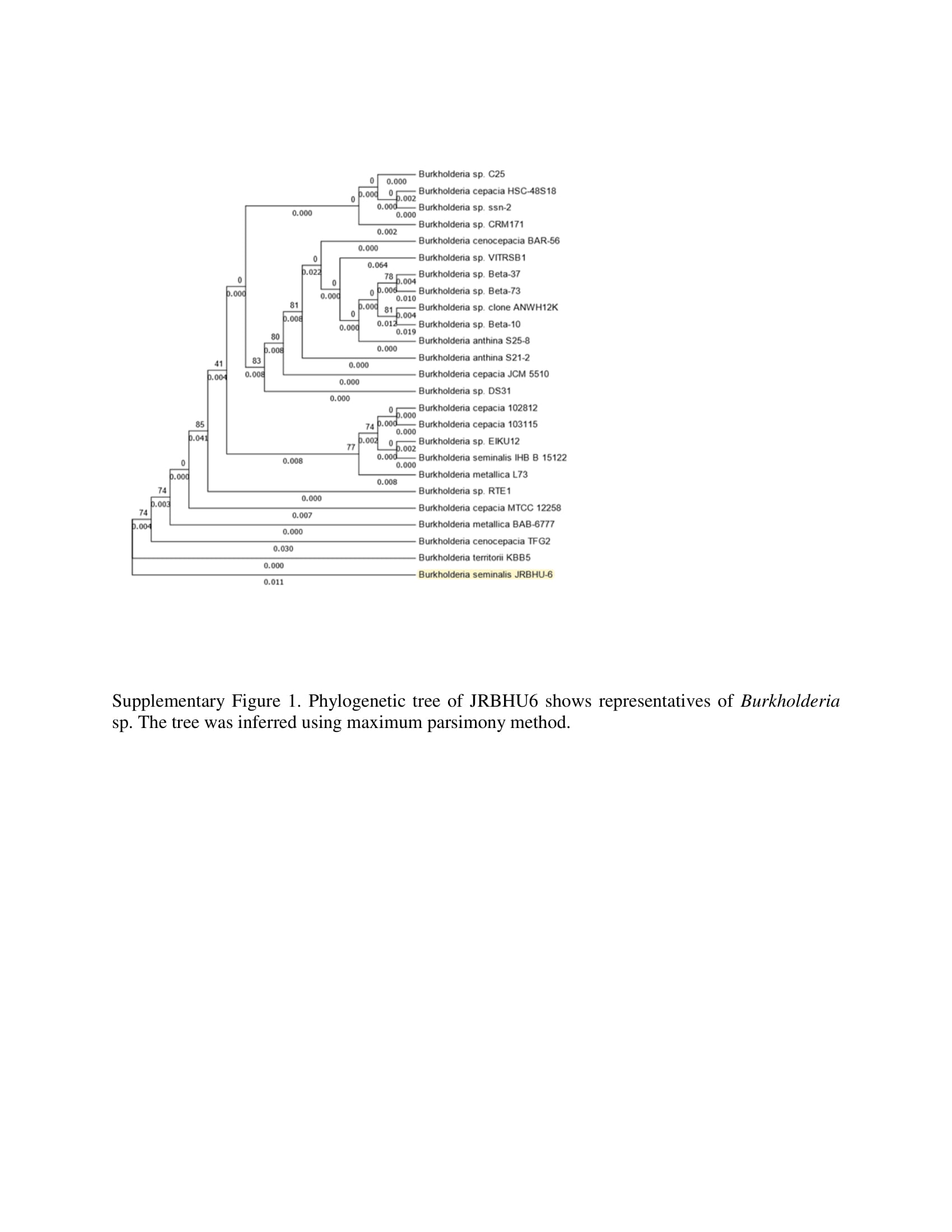

Supplement: Supplementary file 1 [file Image_1.jpg]

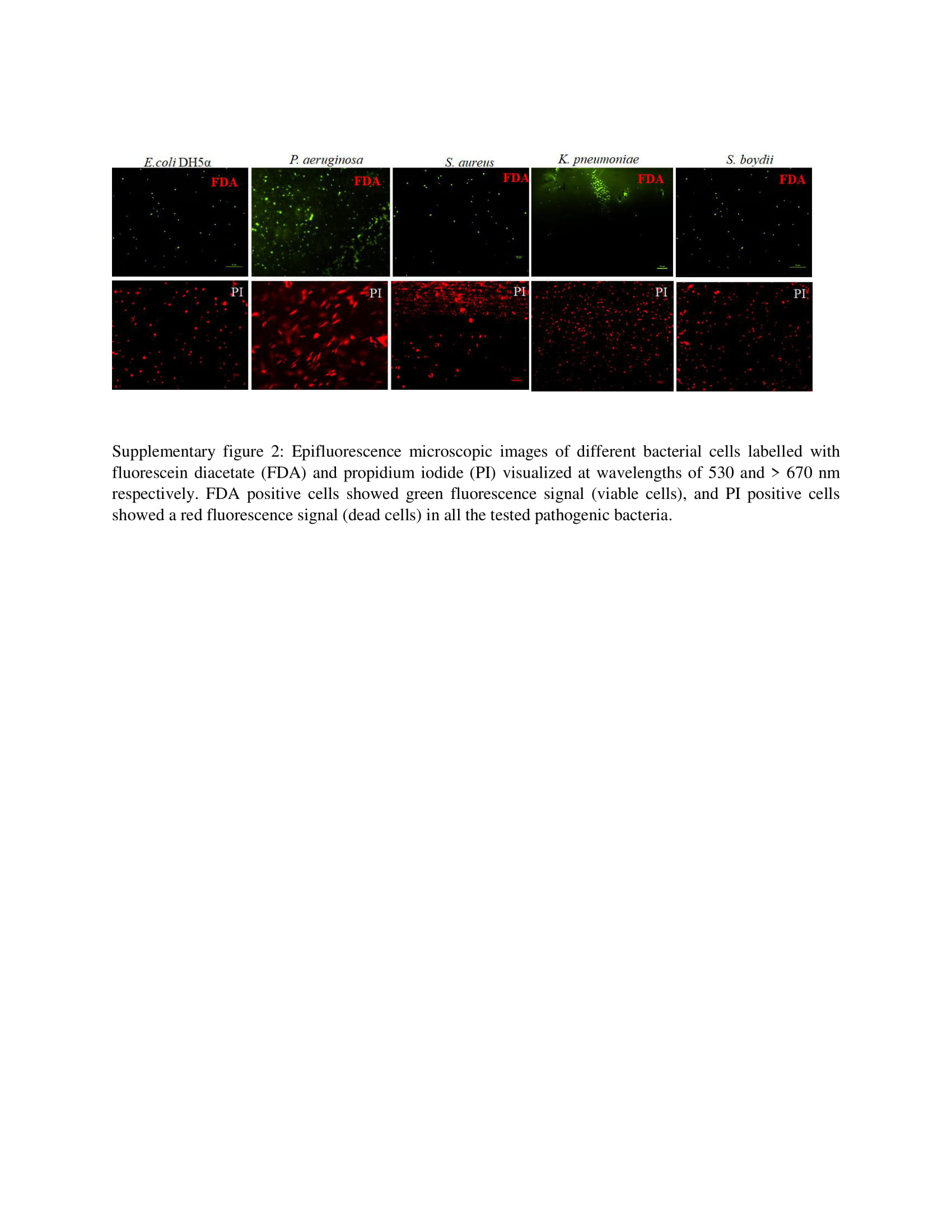

Supplement: Supplementary file 2 [file Image_2.jpg]
